# Supplementary material for: Integration of a vertical voluntary medical male circumcision program into routine health services in Zimbabwe: A solution for sustainable HIV prevention
Source: PLOS Glob Public Health. 2025 Jul 10;5(7):e0003757. doi: 10.1371/journal.pgph.0003757 (PMC12244533; doi:10.1371/journal.pgph.0003757)
Supplement: S3 Table — (DOCX) [file pgph.0003757.s003.docx]

**S3 Table. District Task Team composition**

| **Type** | **Organization/ Program** | **Roles** |
| --- | --- | --- |
| Community | Churches, local government | Pastor, Village Chief, Councillor |
| Facility | MoHCC | Registered General Nurse, Pharmacist |
| District | MoHCC, Ministry of Primary & Secondary Education, local government | District Medical Officer, District Nursing Officer, District Health Promotion Officer, District Health Services Administration Officer, District AIDS Coordinator, District Health Information Officer, District Schools Inspector, District Development Coordinator |
| Provincial | MoHCC | Provincial Medical Director, Provincial Nursing Officer, Provincial Maternal and Child Health Officer, Provincial VMMC Officer, Provincial Health Promotion Officer |
| National |  | AIDS and TB Program Officer, TB Officer, Data & Research Officer, HIV Prevention Coordinator, Program Officer, Monitoring & Evaluation Officer |
| Implementing/ technical partners | CHAI, PSH, ZIM-TTECH, ZACH, ZiCHIRe | Management Development Specialist, Analyst, Deputy Director, HIV Prevention Manager, VMMC Demand Creation Manager |
