# Supplementary material for: Integration of a vertical voluntary medical male circumcision program into routine health services in Zimbabwe: A solution for sustainable HIV prevention
Source: PLOS Glob Public Health. 2025 Jul 10;5(7):e0003757. doi: 10.1371/journal.pgph.0003757 (PMC12244533; doi:10.1371/journal.pgph.0003757)
Supplement: S4 Table — (DOCX) [file pgph.0003757.s004.docx]

**S4. National Task Force composition**

| **Type** | **Organization/ Program** | **Roles** |
| --- | --- | --- |
| National | MoHCC, National AIDS Council | AIDS and TB Program Officer, TB Officer, Data & Research Officer, HIV Prevention Coordinator, Training Officer |
| Provincial | MoHCC | Provincial Medical Director, Provincial Epidemiology and Disease Control Officer, Provincial Maternal and Child Health Officer |
| Implementing & technical partners, donors | PSH, ZIM-TTECH, WHO, CHAI, BMGF, PEPFAR (CDC, USAID), Chemonics | Director, Deputy Program Director, Program Manager, National Professional Officer, Senior Analyst, Country Liaison, HIV Prevention Manager |
