# Supplementary material for: Integration of a vertical voluntary medical male circumcision program into routine health services in Zimbabwe: A solution for sustainable HIV prevention
Source: PLOS Glob Public Health. 2025 Jul 10;5(7):e0003757. doi: 10.1371/journal.pgph.0003757 (PMC12244533; doi:10.1371/journal.pgph.0003757)
Supplement: S5 Table — (DOCX) [file pgph.0003757.s005.docx]

**S5 Table: Examples of localized integration and sustainability definitions**

| Term | Definition |
| --- | --- |
| Sustainability | The continuity of quality VMMC services as part of a comprehensive HIV prevention strategy and recognition of VMMC as an essential service, which must be available in all health institutions at all times with full ownership by all stakeholders. |
| Integration | The recognition of VMMC as part of the package of essential core health services at primary and secondary levels of care which is offered to all eligible clients, together with other health services, at an affordable cost, efficiently utilizing available resources. |
