# Supplementary material for: Integration of a vertical voluntary medical male circumcision program into routine health services in Zimbabwe: A solution for sustainable HIV prevention
Source: PLOS Glob Public Health. 2025 Jul 10;5(7):e0003757. doi: 10.1371/journal.pgph.0003757 (PMC12244533; doi:10.1371/journal.pgph.0003757)
Supplement: Attachment — Submitted filename: Reviewed Document.25Aug24_VMMC_integration_Zim.doc [file pgph.0003757.s008.doc]

**Reviewer’s Report**

**Title:** Sustainable integration of a vertical voluntary medical male circumcision program into routine health services in Zimbabwe: A mixed methods evaluation of a participatory change intervention

**Comment:** The title aligns itself with the Journal and focus on public health, and it appropriately conveys the articles focus and study area. However**, a title could** be refined further: the term sustainable should be removed at the start of the sentence, unless the authors are confident that the program will be sustainable in future, and a mixed methods approach should be taken to methodology section and hence misplaced.

**Suggested title:** Integration of a vertical voluntary medical male circumcision program into routine health services in Zimbabwe: Solution for a sustainable HIV Prevention

# Abstract: the abstract clearly and accurately reflects the content in summary. However, it can be shortened further to attain a concise version:

**Suggested Abstract**

*The article presents information on the importance of integrating and sustaining health programs and forming equitable partnerships in Zimbabwe with the focus on integrating of the vertical Voluntary Medical Male Circumcision (VMMC) into the routine health services. The paper further describes a bottom-up process of sustainable integration of the VMMC program into routine health services, an approach that assumed to empower local stakeholders. The study derives its methodology by the use of a mixed methods design with the focus on qualitative and quantitative analysis and survey assessment. The study shows a reduction in funding stability, however, notably, a significant increase in communications, program adaptation, and organizational capacity was recorded. Therefore, the paper concludes that compared to* *traditional top-down change initiatives, the participatory approach to integration was an effective way of addressing specific VMMC challenges at the district level whilst maintaining management and oversight at provincial and national levels. And lastly, in other health programs especially, the low- and middle-income countries seeking to integrate and sustain health services at subnational levels, the paper recommends that the diagonal, bottom-up model to promote local leadership development and health system strengthening is a better sustainable approach.*

**General comments**

1. *The paper generally addresses the comprehensive aspect of the requirements by the journal, however, there is need for few edits to suit the journal aspirations.*
2. Only minor grammatical adjustments and phrasing refinements are suggested to improve clarity and readability.
3. The abstract accurately reflects the content.
4. *Need to take the mixed methods to the methodology section.*
5. *The use of “We” in the paper should be avoided and probable, rephrased to read, “the team or the Authors”.*
6. *There is need to clearly bring out probable VMMC measures that will be sustainable once donor supported activities are withdrawn or donor program ends.*
7. *The models are clearly illustrated and applied in this paper. The tables are provided outlining the sustainable efforts that exist. However, there is need to recast some of these efforts and provide a unique approach from what exists to a more proactive approach that would result into more desirable sustainable VMMC integration into routine systems.*
8. *Specific insights on how the vertical VMMC program would be integrated bearing in mind of the limited budget of the Government taking into considerations the avoidance to stretch the current budget. A few questions, acceptability on program integration into routine is one thing, and implementation of it for sustainability might mean another thing, how then do we strike the balance? Is Zimbabwe at local levels, district to be precise, capable of rising resources through Income Generating activities (IGA) such as sport Galas, to fund VMMC integration into routine health services in future without stretching the limited budget, and how are health workers avoid the donor dependence and change their work attitude to embrace this integration for a sustainable VMMC program in future, and in this regard, the authors should consider Behavioral trainings and mentorship, a solution to negative attitude by health workers towards VMMC integration into routine once resources are no longer forth coming. And once these are addressed, this paper has potential to replicate its findings to other parts of the country if not the continent as a whole.*
9. *It would be nice to make recommendation after the conclusion for possibility of policy consideration by Government of Zimbabwe*
10. *References are well arranged* *and citations align with the references provided.*

***Reviewer’s Decision***

1. Based the observations and suggested refinements to improve clarity and readability, the paper meets the standards and therefore, **Publish Only After Minor Revision***.*
