# Supplementary material for: Integration of a vertical voluntary medical male circumcision program into routine health services in Zimbabwe: A solution for sustainable HIV prevention
Source: PLOS Glob Public Health. 2025 Jul 10;5(7):e0003757. doi: 10.1371/journal.pgph.0003757 (PMC12244533; doi:10.1371/journal.pgph.0003757)
Supplement: Attachment — Submitted filename: 27Jan25Response to Reviewers.docx [file pgph.0003757.s010.docx]

Dear PLOS Global Public Health Editors and Reviewers:

Thank you for engaging so closely with our work and for your valuable feedback on our manuscript. Please find responses to each point raised by the editor and reviewers below. We hope these responses ensure that the revised manuscript now fully meets the journal’s publication criteria.

*In particular, please ensure that the following criteria is met:*

- *Experiments, statistics, and other analyses are performed to a high technical standard and are described in sufficient detail.*

We have included additional detail to describe the statistics and analyses that we used.

*In addition, clarifying the research question will help ensure the following criteria is fully and clearly met:*

- *The study presents the results of original research.*

The research question our study answered was ‘What were the contributions of the OPTIMISE project to the integration of VMMC into primary health services within intervention districts in Zimbabwe?’

Journal Requirements:

1. *Figure 3: please (a) provide a direct link to the base layer of the map (i.e., the country or region border shape) and ensure this is also included in the figure legend; and (b) provide a link to the terms of use / license information for the base layer image or shapefile.*
a) <https://commons.wikimedia.org/wiki/File:Blank_Zimbabwe_Map.svg>
b) <https://commons.wikimedia.org/wiki/Commons:Reusing_content_outside_Wikimedia>

SUGGESTED REVISIONS FROM EDITOR (including both major and minor revisions).

A. GENERAL
   *1. Authors are requested to proof-read the final version of the submitted manuscript to ensure clear sentence and communication, avoiding, among others, incomplete details in tables, etc.*

We have proof-read the final version to ensure sentences are clear and all tables have complete details.

2. *At times, it is not clear if this is a "lessons-learned" paper that fully describes the process of implementing and transitioning from vertical to horizontal programming for VMMC OR if this is a research paper, guided by a clear research question, that evaluates the transition.*

The research question our study answered was ‘What were the contributions of the OPTIMISE project to the integration of VMMC into primary health services within intervention districts in Zimbabwe?’

B. ABSTRACT.
*1. Authors are encouraged to summarize results in more detail and include them in the abstract.*

We replaced these results:

“In all five pilot districts we facilitated the transition of VMMC into the government’s district administration, resulting in a locally owned and managed program, while also strengthening individual and team capacity. We observed improvements across all World Health Organization health system building blocks, suggesting that the intervention strengthened the overall health system.”

with the following:

“Over a two-year period, Task Teams in all five intervention districts successfully integrated the VMMC program by reducing barriers and leveraging opportunities across other parts of the health system, leading to increased program capacity and sustainability. Key outcomes impacted WHO health system building blocks, including enhanced leadership and governance, improved service delivery through better access and acceptability, an expanded health workforce through training, more efficient use of medical technologies, improved data quality, and the mobilization of local funds to support program financing and sustainability.”

*2. Authors are encouraged to include some conclusions.*

We replaced this:

“Compared to traditional top-down change initiatives, the participatory approach to integration was an effective way of addressing specific VMMC challenges at the district level whilst maintaining management and oversight at provincial and national levels.”

with the following:

“The integration of VMMC into district health systems resulted in significant improvements in program sustainability, leadership, service delivery, and workforce capacity. By

, fostering local ownership, and mobilizing resources, the project demonstrated a successful model for scaling and sustaining VMMC services.”

and kept this part in as part of the conclusion:

“Other health programs in low- and middle-income countries seeking to integrate and sustain health services at subnational levels should consider this diagonal, bottom-up model to promote local leadership development and health system strengthening.”

*3. Consider adding "program" after VMMC (third sentence).*

We deleted “the” at the beginning of the third sentence, so that it now reads: “Voluntary Medical Male Circumcision (VMMC) in Zimbabwe is a large vertical HIV prevention program primarily funded through development assistance for health.”

C. INTRODUCTION
*1. Need to have focus around the issue being researched. Some of the information presented distract from the focus of the paper. For instance, some of the details of the implementation of the project can be summarized more succinctly as to give context to the research question but not to provide details that are not directly related to the research question.*

We have cut down implementation details to describe the project more succinctly so as to focus on context related to the research question.

*2. Authors are encouraged to briefly explain the LEAD framework and the OPTIMISE project in this section, not in the methods sections.*

We have moved explanation of the LEAD Framework and OPTIMISE project from the methods section to the introduction.

*3. Authors should provide a sub-heading that briefly describes Zimbabwe, its provinces and districts, and the structure of the health system, and demographics of the population (at least int he selected districts compared to national averages) for the characteristics that are relevant to this study (e.g., males, 15-29 years old), to give reader context on the work presented. For instance, Page 4 states that 63 districts are government run, one may asked "how many districts are there in total?"*

Under a health system subheading, we have added details to describe Zimbabwe, its provinces and districts, including the structure of the health system. Demographics of the population have been added under the intervention area and study population subheading. There are 63 districts in total throughout the country, and all are government run, but the VMMC program is not fully owned and operated in all districts. These details have been added to the manuscript.

D. METHODS
*1. Please clearly specify what the research question is.*

The research question our study answered was: What were the contributions of the OPTIMISE project to the integration of VMMC into primary health services within intervention districts in Zimbabwe?’

*2. Explain and describe, in a different sub-heading, the study population.*

Details about the study population were added under the intervention area and study population sub-heading.

*3. Authors state that this work used a mixed-methods approach; however, no description of the methodology is done. What type of mixed-methods study design was used (for instance quant-to-qual, concurrent, etc.)?.*

We used a concurrent design, which combined the gathering of qualitative and quantitative data from the district work plans as well as administering a quantitative survey at midline and endline.

*4. The methods section is mostly descriptive of work done. Some of this information should be in the introduction. The actual methods are not described in sufficient detail.*

We have moved the description of work done to the introduction and added more detail on the work plan data collection and survey administered in the methods section under the subheading of ‘Data sources and analysis’. The description includes how we determined integration, through the comparison of baseline and endline workplan data, and supplemented these findings through calculating the mean midline and endline survey results for each of the eight survey domains and performing 2 sample t tests.

*5. Authors need to provide a clear set of eligibility criteria both at the programmatic level (i.e., which districts/program were included - some of this is presented, needs to be highlighted, structured and focused) as well as the individual level (for the surveys and other research procedures).*

We have added more structured and focused detail around eligibility for the intervention districts and as well as for the survey participants under the intervention areas and study population subheading.

*6. Authors need to specify sample size calculations, if any. If no calculations, explain how many people were included and provide rationale for sample size. Also, explain sampling methodology.*

We included 54 respondents in the midline survey and 59 respondents in the endline survey and used convenience sampling for the survey. Details have been added to the revised manuscript.

*7. Authors need to describe the qualitative methodology.*

We conducted a document review of district work plans, which included qualitative and quantitative indicators devised by the district teams that were then reported at baseline and endline.

*8. Authors need to describe the quantitative methodology.*

In addition to the qualitative document review, the Program Sustainability Assessment Tool was a quantitative survey.

*9. Authors need to better describe data collection, data storage and manipulation, and data analysis plans, including a priori defined levels of significance, as appropriate.*

We have added additional detail concerning data collection, storage, analysis, and levels of significance.

*10. Authors should better describe the PSAT tool used.*

Additional detail on the PSAT tool has been provided.

E. RESULTS
*1. Authors are encouraged to describe better table results in the narrative of the results.*

We have amended the narrative to better describe the table results.

F. DISCUSSION
*1. Authors are encouraged to consider the implication of using autoclaves instead of disposable surgical materials. For instance, are there the required systems in place to avoid contamination (e.g., appropriate training, adequate operating and maintenance protocols, etc.?*

We have addressed autoclaving and the benefits of reusable surgical instruments over disposable materials in this section along with the training and operating and maintenance protocols that accompany autoclaving.

*Suggest reviewing the sentence "we set out to integrate the VMMC program into routine services" in light of the research question being answered by this project - for instance, is the goal of this paper to describe the integration of the services or the evaluation of such integration?*

The goal of the paper was to *evaluate* the integration of VMMC into routine services.

*I suggest authors to not include the link to the LEAD framework guide in the manuscript, but rather add a citation and then, in the citation, provide all the information necessary using standard citation styles for the reader to access the guide.*

We have incorporated this suggested edit.

*4. Suggest adding citation to the LEAN/Six Sigma reference.*

We had also added this citation.

[Note: HTML markup is below. Please do not edit.]

**Reviewers' comments**:

**Reviewer #1**: *Your work set out to address a very important area on integration and sustainability of VMMC in Zimbabwe and I found reading it very interesting.*

We thank Reviewer #1 for this encouraging and supportive comment. Our responses to specific feedback and recommendations are detailed below.

Major Issues:
*1) I noted that from the outset the criteria for the selection of the provinces and districts was given among others as support by donors/other implementing partners, and accessibility by road. Regarding the former, Nyanga in Manicaland (Table 1) was the only one among the 5 districts lacking in this area. One would have expected a clarification as to why Nyanga (with this gap) was in the inclusion criteria. I was happy to see that Nyanga got quite innovative identifying local funds e.g. for vehicle servicing and fueling. For purposes of replication and lessons learnt, it would be useful to clarify what this "local funding" was. On the other hand, the value of accessibility by road as a selection criteria did not come out clearly.*

Nyanga was included as an intervention district because it had limited donor support, no assigned implementing partners, and was accessible by road from Harare. All five districts are easily accessible by road. This selection criterion is explicitly documented in the revised paper.

The local funding identified by Nyanga came from the government and user fees, which all districts have. The District Health Executive can make decisions on how to use these funds, and in this case, it prioritized some of its funding towards improving data quality.

*2) I also observed that Hwange successfully integrated the VMMC into their District Platform which is commendable. Is there any information as to why other districts could not do the same.*

VMMC was integrated into all five district platforms. We have clarified this in the narrative to make this more explicit.

3) What can the other four districts learn on how to access government funds from Matobo? could more light be shed on this?

Districts are not able to control allocations and disbursements from the Government of Zimbabwe (GoZ), but they can try to efficiently use what is disbursed. After a district receives funds from the GoZ, they make decisions on how to use the funds received based on integrated annual plans and emerging priorities. Due to the vertical approach to VMMC and limited involvement in planning, budgeting and financial management processes, the DHE was not aware of the gaps in VMMC funding. However, after engagement and the inclusion of VMMC on the agenda, the Matobo DHE became aware of the gaps and started allocating resources towards outreach, fuel, and mentoring. A lesson that other districts can learn from Matobo is that the DHE should be involved and empowered to plan, budget, monitor, and manage the financial processes across all health programs.

*4) Finally, your study has several limitations and I am happy to see you have pointed them out. Unfortunately, I think all those limitations impact negatively on the reproducibility/replication of this important study.*

Reflecting on the findings of this study and the major gap they are intended to address, we see important and innovative ways that our work supports a much needed ‘localization’ agenda. Our claim is that the global health research and implementation community needs to embrace localization more fully. Intervention designs of this sort are not formally ‘experimental’ and therefore cannot fully meet the formal requirements of research replication. However, the *methodology* is replicable and can be used to pursue program integration in other local/specific contexts and settings.

Minor Issue-
*1) Some Tables appear to have incomplete entries, for example some information for Hwange and Matobo in Table %*

We have reviewed the tables and filled in missing data for Hwange and Matobo.

*2) I believe in under your References, citations accessed from https should indicate the date accessed.*

We have added the date accessed to any citations with https that were missing the date cited.

**Reviewer #2**:

**Title:** Sustainable integration of a vertical voluntary medical male circumcision program into routine health services in Zimbabwe: A mixed methods evaluation of a participatory change intervention

***Comment:*** *The title aligns itself with the Journal and focus on public health, and it appropriately conveys the articles focus and study area. However****, a title could*** *be refined further: the term sustainable should be removed at the start of the sentence, unless the authors are confident that the program will be sustainable in future, and a mixed methods approach should be taken to methodology section and hence misplaced.*

***Suggested title:*** *Integration of a vertical voluntary medical male circumcision program into routine health services in Zimbabwe: Solution for a sustainable HIV Prevention*

Thank you for this helpful suggestion. We have changed the title accordingly: removing reference to ‘mixed methods’ and moving ‘sustainable’ to the subtitle.

***Abstract:*** *the abstract clearly and accurately reflects the content in summary. However, it can be shortened further to attain a concise version:*

**Suggested Abstract**

*The article presents information on the importance of integrating and sustaining health programs and forming equitable partnerships in Zimbabwe with the focus on integrating of the vertical Voluntary Medical Male Circumcision (VMMC) into the routine health services. The paper further describes a bottom-up process of sustainable integration of the VMMC program into routine health services, an approach that assumed to empower local stakeholders. The study derives its methodology by the use of a mixed methods design with the focus on qualitative and quantitative analysis and survey assessment. The study shows a reduction in funding stability, however, notably, a significant increase in communications, program adaptation, and organizational capacity was recorded. Therefore, the paper concludes that compared to* *traditional top-down change initiatives, the participatory approach to integration was an effective way of addressing specific VMMC challenges at the district level whilst maintaining management and oversight at provincial and national levels. And lastly, in other health programs especially, the low- and middle-income countries seeking to integrate and sustain health services at subnational levels, the paper recommends that the diagonal, bottom-up model to promote local leadership development and health system strengthening is a better sustainable approach.*

Thank you for your recommendation. We have revised the abstract as suggested while also responding to comments from the other reviewer.

**General comments**

1. *The paper generally addresses the comprehensive aspect of the requirements by the journal, however, there is need for few edits to suit the journal aspirations.*
2. *Only minor grammatical adjustments and phrasing refinements are suggested to improve clarity and readability*.

We have made minor grammatical adjustments and phrasing refinements as suggested.

3. *The abstract accurately reflects the content.*

In line with your recommendation, we have revised the abstract so that it accurately reflects the content.

4. *Need to take the mixed methods to the methodology section.*

We removed ‘mixed methods’ from the title and elaborated on this approach in the methods section.

5. *The use of “We” in the paper should be avoided and probable, rephrased to read, “the team or the Authors”.*

We have replaced “we” with “the team”, ‘research team”, “the study team”, or “the authors.”

6. *There is need to clearly bring out probable VMMC measures that will be sustainable once donor supported activities are withdrawn or donor program ends.*

In response to your guidance, we make clear in the revised paper which VMMC measures have been impacted by the LEAD intervention. Longer-term inferences can be made from the evidence. Sustainability as defined by WHO exists along a continuum and involves increased responsibility by local health authorities, domestic financing, an adequate health workforce and supplies and equipment, quality data collection, management, analysis and use, and increased access and a reorientation of service delivery. All aspects of WHO-defined sustainability were demonstrated by our intervention districts. A key driver of sustainable delivery of outcomes will be sustainable practice of the LEAD approach to problem-solving. This is in the hands of local health systems managers at all levels and should be monitored.

7. *The models are clearly illustrated and applied in this paper. The tables are provided outlining the sustainable efforts that exist. However, there is need to recast some of these efforts and provide a unique approach from what exists to a more proactive approach that would result into more desirable sustainable VMMC integration into routine systems.*

We suspect there may some slight misunderstanding here regarding data presented in the tables. The data at baseline shows the existing sustainable efforts at baseline (little to nothing), while the endline data shows the changes that the district teams proactively made to sustainably integrate VMMC into routine systems.

8. *Specific insights on how the vertical VMMC program would be integrated bearing in mind of the limited budget of the Government taking into considerations the avoidance to stretch the current budget. A few questions, acceptability on program integration into routine is one thing, and implementation of it for sustainability might mean another thing, how then do we strike the balance? Is Zimbabwe at local levels, district to be precise, capable of rising resources through Income Generating activities (IGA) such as sport Galas, to fund VMMC integration into routine health services in future without stretching the limited budget, and how are health workers avoid the donor dependence and change their work attitude to embrace this integration for a sustainable VMMC program in future, and in this regard, the authors should consider Behavioral trainings and mentorship, a solution to negative attitude by health workers towards VMMC integration into routine once resources are no longer forth coming. And once these are addressed, this paper has potential to replicate its findings to other parts of the country if not the continent as a whole.*

The reviewer makes an excellent point concerning the challenges of sustaining the VMMC program when there is finite government funding. There is ongoing advocacy at national levels to allocate more government funding to HIV prevention. At the same time, there are efficiencies or changes to the way the program operates that the districts implemented and which can reduce costs and save limited funding. Behavioral trainings and mentorship might be a partial counter to negative attitudes but, rather, as we have tried to highlight, increased autonomy around decision-making of local budgets and operations can contribute to increased motivation on the part of health workers. These have been coupled with pilots to move from fee-for-service to results-based financing, as well as sub-granting of funds to subnational entities.

9. *It would be nice to make recommendation after the conclusion for possibility of policy consideration by Government of Zimbabwe*

We have included policy considerations for the Government of Zimbabwe in the conclusion section.

10. *References are well arranged* *and citations align with the references provided.*

Thank you for this valuable feedback.

This concludes our response letter.

Sincerely,

Amanda Marr Chung, DrPH, MPH
