# Supplementary material for: Integration of a vertical voluntary medical male circumcision program into routine health services in Zimbabwe: A solution for sustainable HIV prevention
Source: PLOS Glob Public Health. 2025 Jul 10;5(7):e0003757. doi: 10.1371/journal.pgph.0003757 (PMC12244533; doi:10.1371/journal.pgph.0003757)
Supplement: Attachment — Submitted filename: PLOS GPH Response Letter - May 25.docx [file pgph.0003757.s011.docx]

**Response Letter PC Edits**

Dear Dr. Reina Ortiz and *PLOS Global Public Health* Reviewers:

Thank you for engaging so closely with our work and for your valuable feedback on our manuscript. Please find responses to each point raised by the editor and reviewers below. We hope these responses ensure that the revised manuscript now fully meets the journal’s publication criteria.

Journal Requirements:

*Please review your reference list to ensure that it is complete and correct. If you have cited papers that have been retracted, please include the rationale for doing so in the manuscript text, or remove these references and replace them with relevant current references. Any changes to the reference list should be mentioned in the rebuttal letter that accompanies your revised manuscript. If you need to cite a retracted article, indicate the article’s retracted status in the References list and also include a citation and full reference for the retraction notice.*

Thank you for your careful review of the reference list. We have corrected and updated some references, removed duplicate entries of the first two references, and ensured that we have removed any retracted papers. Papers that are no longer available online and that we have removed from the reference list include the following:

1. World Health Organization. Integrated health services -what and why? Technical Brief No. 1. 2008 May;10. And this reference
2. USAID. Local Partner Transition | Global Health [Internet]. U.S. Agency for International Development. 2023 [cited 2023 Nov 22]. Available from: https://www.usaid.gov/global-health/health-areas/hiv-and-aids/technical-areas/local-partner-transition

There are also separate references for the Accra Agenda for Action and the Paris Declaration on Aid Effectiveness.

Additional Editor Comments (if provided):

*1. Rephrase the "current state of VMMC programs" section in the introduction as it likely is not reflective of "current" state but of "current state" at the time the introduction was written. Give it a title that is reflective of the content discussed there.*

We have revised the heading from “current state of VMMC programs” to “status of VMMC programs.” We have also revised some of the text in the subsection to reflect the current state of funding following the recent withdrawal of US development assistance for health.

*2. Ensure that all references to PEPFAR's policies are current.*

We have ensured all references to PEPFAR’s policies are current, including adding another document entitled: ‘PEPFAR’s Five-year Strategy: Fulfilling America’s Promise to End the HIV/AIDS Pandemic by 2030’. The current US administration has not updated these documents as of May 2025.

*3. This sentence needs to be rephrased to increase clarity: "In 2020, the MoHCC sought support from University of California, San Francisco (UCSF)’s LEAD team, complementing the efforts of CHAI and Population Solutions for Health (PSH), in transitioning the VMMC program to sustainability on national and subnational levels." Previously, the authors mentioned that CHAI developed a VTAD. It may be helpful to keep events in chronological order and tied together. A figure showing steps in the process taken "thus far" (i.e., before the study) to ensure integration, transition to a horizontal approach, and sustainability would be helpful.*

Thank you for this suggestion. We have revised this sentence to improve clarity as follows:

“To complement the ongoing efforts of CHAI and Population Solutions for Health (PSH) in transitioning the VMMC program to sustainability, the MoHCC sought support from University of California, San Francisco (UCSF)’s LEAD team in 2020.”

We also created a figure to show steps in the sustainability transition process and the other stakeholders involved.

Fig 1. Steps in the VMMC sustainability process in Zimbabwe

*4. LEAD framework. First describe the LEAD framework, then describe the adaptations.*

Thank you for this suggestion. We have revised the manuscript to describe the adaptations that we made after we first described the LEAD Framework.

*5. Research question: specify what "this intervention" refers to.*

We have specified the LEAD Framework as the intervention in the research question.

*6. "Health system" subsection in the methods section can be included in the "Study Population" section.*

Thank you for this suggestion. As recommended, we have repositioned this subsection under the Study Population section.

*7. Concurrent mixed methods study design usually is described as either KII or FGDs (qualitative) used in conjunction with surveys or similar quantitative. Ensure that the right methodology is described or provide evidence that document review has been used in concurrent mixed methods study design.*

We have removed mention of concurrent mixed methods study design and described the methods we employed as a review of qualitative and quantitative data from work plans combined with a quantitative survey.

*8. Describe which quantitative and qualitative indicators were used.*

The quantitative and qualitative indicators vary depending on what the districts selected and can be seen in Tables 4-7. We have clarified in the text that these indicators were co-created by the district and research teams.

*9. Authors mentioned that data was scanned - indicate how data was extracted into data base for data analysis. Was date entered manually? By how many investigators? detail data cleaning procedures, as applicable.*

We have revised the manuscript to indicate that we took photos of the post workshop evaluation forms and then manually reviewed and analyzed the data rather than extracting them into a database where data was entered manually and cleaned.


[Note: HTML markup is below. Please do not edit.]

Reviewers' comments:

Reviewer's Responses to Questions

**Comments to the Author**

1. If the authors have adequately addressed your comments raised in a previous round of review and you feel that this manuscript is now acceptable for publication, you may indicate that here to bypass the “Comments to the Author” section, enter your conflict of interest statement in the “Confidential to Editor” section, and submit your "Accept" recommendation.

Reviewer #1: All comments have been addressed

Reviewer #3: All comments have been addressed

2. Does this manuscript meet PLOS Global Public Health’s [publication criteria](https://track.editorialmanager.com/CL0/https:%2F%2Fjournals.plos.org%2Fglobalpublichealth%2Fs%2Fcriteria-for-publication/1/010f019632043359-5156fea9-ef46-4d84-9cd3-290a1f9f580d-000000/fJGrYLzxWyTGSucZVFEyme3QH9EC_mcacy3ItEpItgc=206)? Is the manuscript technically sound, and do the data support the conclusions? The manuscript must describe methodologically and ethically rigorous research with conclusions that are appropriately drawn based on the data presented.

Reviewer #1: Yes

Reviewer #3: Yes

3. Has the statistical analysis been performed appropriately and rigorously?

Reviewer #1: I don't know

Reviewer #3: N/A

4. Have the authors made all data underlying the findings in their manuscript fully available (please refer to the Data Availability Statement at the start of the manuscript PDF file)?

The [PLOS Data policy](https://track.editorialmanager.com/CL0/https:%2F%2Fjournals.plos.org%2Fglobalpublichealth%2Fs%2Fdata-availability/1/010f019632043359-5156fea9-ef46-4d84-9cd3-290a1f9f580d-000000/-Rn4me6clSIH59PJHWfg81TO7Vi9NJialr6CIQmjtVA=206) requires authors to make all data underlying the findings described in their manuscript fully available without restriction, with rare exception. The data should be provided as part of the manuscript or its supporting information, or deposited to a public repository. For example, in addition to summary statistics, the data points behind means, medians and variance measures should be available. If there are restrictions on publicly sharing data—e.g. participant privacy or use of data from a third party—those must be specified.

Reviewer #1: Yes

Reviewer #3: Yes

5. Is the manuscript presented in an intelligible fashion and written in standard English?

PLOS Global Public Health does not copyedit accepted manuscripts, so the language in submitted articles must be clear, correct, and unambiguous. Any typographical or grammatical errors should be corrected at revision, so please note any specific errors here.

Reviewer #1: Yes

Reviewer #3: Yes

6. Review Comments to the Author

Please use the space provided to explain your answers to the questions above. You may also include additional comments for the author, including concerns about dual publication, research ethics, or publication ethics. (Please upload your review as an attachment if it exceeds 20,000 characters)

Reviewer #1: N/A

Reviewer #3: The authors have thoroughly addressed comments raised by the editor and reviewers, and the revised manuscript seems suitable for publication now.

7. PLOS authors have the option to publish the peer review history of their article ([what does this mean?](https://track.editorialmanager.com/CL0/https:%2F%2Fjournals.plos.org%2Fglobalpublichealth%2Fs%2Feditorial-and-peer-review-process%23loc-peer-review-history/1/010f019632043359-5156fea9-ef46-4d84-9cd3-290a1f9f580d-000000/-R4xNpCnk3Rh6k1CMJg2MjToxA2jQwf1zloxXOGDFDA=206)). If published, this will include your full peer review and any attached files.


**Do you want your identity to be public for this peer review?** If you choose “no”, your identity will remain anonymous but your review may still be made public.


For information about this choice, including consent withdrawal, please see our [Privacy Policy](https://track.editorialmanager.com/CL0/https:%2F%2Fwww.plos.org%2Fprivacy-policy/1/010f019632043359-5156fea9-ef46-4d84-9cd3-290a1f9f580d-000000/9rrogCl1Sc3u0vFZOLEFOomrq6NAhnPnimeXNxNtuJU=206).

Reviewer #1: No

Reviewer #3: **Yes:** Mujahid Abdullah

[NOTE: If reviewer comments were submitted as an attachment file, they will be attached to this email and accessible via the submission site. Please log into your account, locate the manuscript record, and check for the action link "View Attachments". If this link does not appear, there are no attachment files.]

While revising your submission, please upload your figure files to the Preflight Analysis and Conversion Engine (PACE) digital diagnostic tool, <https://pacev2.apexcovantage.com/>. PACE helps ensure that figures meet PLOS requirements. To use PACE, you must first register as a user. Registration is free. Then, login and navigate to the UPLOAD tab, where you will find detailed instructions on how to use the tool. If you encounter any issues or have any questions when using PACE, please email PLOS at figures@plos.org. Please note that Supporting Information files do not need this step.

Thank you for this valuable feedback.

This concludes our response letter.

Sincerely,

Amanda Marr Chung, DrPH, MPH
